# Clinical Reports—Being Abstracts from Clinical Lectures

**Published:** 1882-06

**Authors:** Jno. Thad. Johnson

**Affiliations:** Atlanta, Ga., Professor of Principles and Practice of Surgery in Southern Medical College, Atlanta


### CLINICAL REPORTS—BEING ABSTRACTS FROM CLINICAL LECTURES.

BY JNO. THAD. JOHNSON, M.D., ATLANTA, GA.,
Professor of Principles and Practice of Surgery in Southern Medical College, Atlanta.

M. W., colored female, æt. 22, was delivered of a healthy child on January 26th. The child seems to be still in perfect health. The mother now presents a well-marked and abundant crop of syphilitic mucous patches on the vulva, in the vagina and at the upper inner surface of the thighs. The patient confessed to illicit intercourse to within a week of delivery, and again a few days after delivery.

*Remarks.*—The points of interest here are these: This evidently is a case of syphilis in its early secondary stage. Was it contracted before the birth of the child? If so, how has the child escaped the disease? It is generally regarded as certain that the syphilitic mother certainly infects the child. However, may it be that if the disease is contracted very late in gestation, the child may escape, even though the mother breaks out with active syphilis a few days after birth? We know that syphilis is already fully established long before the so-called secondary symptoms appear; indeed, we know it is already a general disease before the primary sore appears. Or, may the child even now be syphilitic, even though it appears healthy? May the disease declare itself years hence? We may answer, here, that the mother, so actively syphilitic, would scarcely bring forth a child with syphilis in a latent form.

Again, if the disease was acquired by the patient subsequent to the birth of her child, see how short was the period between contagion and general eruption. She confesses to intercourse within one week or ten days after delivery. Say, then, it may have been contracted February 1st. We first discover these patches or ulcers on March 1st; they are already of several days duration. Remembering that at least two weeks, usually a longer time, elapses between contagion and the primary lesion, and at least 40 days, usually more, between this and general symptoms, it is difficult to believe the inoculation occurred after confinement. Then, we must think the mother received the disease prior to her delivery; and at so late a date of gestation that the child escaped; or that, more difficult to believe, the child is really diseased and will yet give evidence of it.

I may here properly allude to a couple of cases that came under my observation sometime since, illustrating the long period of incubation possible for syphilis. Two young men, intelligent and truthful, and anxious to give all the facts that might throw light upon their case, had intercourse with the same woman at an interval of but a few hours. Neither was exposed after this. The primary lesion was first observed in one after sixty-seven, and in the other after sixty-nine days. Fournier, I believe, gives the history of a patient whose primary incubation was seventy-two days. I am sure this period or stage of syphilis is longer than is generally suspected.

How shall we treat this patient? We will give mercury. When you are sure your patient has constitutional syphilis, give some form of mercury. But do not administer it until you are sure of your diagnosis. There are two reasons for withholding it until you are sure of your diagnosis: First, if not so assured, you may urge a potent remedy without sufficient cause; second, unless so assured, the remedy may so interfere with, or prevent, the development of the disease, if present, as to leave you in uncertainty and doubt in the future.

There is no better preparation of mercury, for most instances of this disease, than blue mass. It is more certain and less irritating in its action than the prot-iodide. We will give her nine grains per diem, in three doses. We will also order, as a local application, calomel powered upon the eruptions, often cleansing them with plain water.

[Two weeks later the patient was free of all symptoms, and much improved in health and in appearance. The blue pill was continued in one-half the quantity first administered.]

*Fissure of Lower Lip.*—This young man, eighteen years of age, has a fissure of the lower lip, resembling the fissure so often met in the upper lip. In this instance, however, it is not a congenitial deformity. He says that when a child he was profusely salivated, an immense ulcer of the lip was one of the results, and cicatrization left this deep fissure permanent. The edges of the fissure are blended with the gum.

These adhesions were separated, the hare-lip operation made, under ether, and the pin and the interrupted silk suture applied. The case seemed to progress well; but the patient, disregarding instructions, left the city in two or three days; and it is reported that while the improvement was great, the success was not such as otherwise would have been secured.

*Hare-Lip.*—A white girl, 13 years of age, was operated on for single hare-lip. A very slight fissure of the soft palate was also present; this will not be interfered with to-day. The paring of the edges of the lip-fissure were made from above downward. The lower portion of these parings were not severed from the lip, but were stitched together with silk, with the hope that this measure would avoid, to a great extent, the notch that so frequently is left at the margin of the lip by the retraction of the edges of the fissure. The result was all that could have been expected.

*Remarks.*—The special object aimed at in the operation is the prevention of the notch alluded to. It is probably impossible to avoid it to at least a slight extent. The method here adopted, and another to be mentioned, probably best accomplishes this object. This second one is sometimes called the triangular plat, or the triangular operation (a diagram is here made on the board to illustrate it.) The incision (on each side) may be made with knife or scissors, and may be commenced at either extremity of the fissure—that is, just under the nose or at the margin of the lip. It passes outward, slightly toward the cheek, makes an obtuse angle, and then to the other extremity of the fissure. The incision thus leaves a receding angle on each side of the *fissure.* These two angles are some little above the margin of the *lip.* The pin is introduced as usual; and by approximating these two receding angles, the lower extremity of the fissure—that at the margin of the lip—is made to *pout*, so as to obliterate, to a degree, the almost inevitable notch.

This girl should have been operated on when an infant. The cure would have been at least as perfect, the child could have taken its nourishment more eusily, and much mental distress in after life, because of the deformity, thereby avoided.

*Epithelioma.*—A middle-aged colored woman presented, with a small warty growth, partly fissured and ulcerated, on the face three-fourths of an inch below the orbit. It had existed for several years, but recently began to increase in size, became irritable and painful, and fissured or ulcerated easily. The whole growth was excised.

*Remarks.*—This, it can scarcely be doubted, is an incipient epithelioma. Of all methods of removal of these growths that have been proposed and practiced, there is nothing that equals, for most cases, excision by the knife. In some instances, where not enough sound tissue can be found for recovering the cavity left by the removal of the growth, some form of caustic may serve a good purpose by removing the foreign structure, or placing the ulcer in a condition to granulate. Let me say here, too, that in cases of this kind, if there be doubt as to the character of the growth, it is best to operate. How seldom do we see a case in which there was cause to regret the prompt removal of such an interloper! But how often do we have occasion to feel the keenest regret that decided measures were not adopted more promptly!

---

The negro medical college of Nashville graduated a class of eight at the recent commencement.

---

Chian turpentine, as a remedy for cancer, has gone to join the shade of cundurango.

---

It is said that Dr. J. Marion Sims has improved his health by his residence in the south of France.

